# Reducing provider workload while preserving patient safety via a two-way texting intervention in Zimbabwe’s voluntary medical male circumcision program: study protocol for an un-blinded, prospective, non-inferiority, randomized controlled trial

**DOI:** 10.1186/s13063-019-3470-9

**Published:** 2019-07-23

**Authors:** Caryl Feldacker, Vernon Murenje, Scott Barnhart, Sinokuthemba Xaba, Batsirai Makunike-Chikwinya, Isaac Holeman, Mufuta Tshimanga

**Affiliations:** 1 International Training and Education Center for Health (I-TECH), 325 9th Avenue, HMC#359932, Seattle, WA 98104-2499 USA; 2 Department of Global Health, University of Washington, 325 9th Avenue, HMC# 359931, Seattle, WA 98104 USA; 3 International Training and Education Center for Health (I-TECH)/Zimbabwe, Harare, Zimbabwe; 4 Department of Medicine, University of Washington, Box 356420, 1959 NE Pacific Street, Seattle, WA 98195-6420 USA; 5 Zimbabwe Ministry of Health and Child Care, Harare, Zimbabwe; 6 Medic Mobile, 3254 19th Street, Floor Two, San Francisco, CA 94110 USA; 7 Zimbabwe Community Health Intervention Project (ZiCHIRe), Harare, Zimbabwe

**Keywords:** Voluntary medical male circumcision, Zimbabwe, Mobile health, Healthcare delivery innovations, Post-operative follow-up

## Abstract

**Background:**

Surgical male circumcision (MC) safely reduces risk of female-to-male HIV-1 transmission by up to 60%. The average rate of global moderate and severe adverse events (AEs) is 0.8%: 99% of men heal from MC without incident. To reach the 2016 global MC target of 20 million, productivity must double in countries plagued by severe healthcare worker shortages like Zimbabwe. The ZAZIC consortium partners with the Zimbabwe Ministry of Health and Child Care and has performed over 120,000 MCs. MC care in Zimbabwe requires in-person, follow-up visits at post-operative days 2,7, and 42. The ZAZIC program AE rate is 0.4%; therefore, overstretched clinic have staff conducted more than 200,000 unnecessary reviews of MC clients without complications.

**Methods:**

Through an un-blinded, prospective, randomized, controlled trial in two high-volume MC facilities, we will compare two groups of adult MC clients with cell phones, randomized 1:1 into two groups: (1) routine care (control group, *N* = 361) and (2) clients who receive and respond to a daily text with in-person follow up only if desired or if a complication is suspected (intervention group, *N* = 361). If an intervention client responds affirmatively to any automated daily text with a suspected AE, an MC nurse will exchange manual, modifiable, scripted texts with the client to determine symptoms and severity, requesting an in-person visit if desired or warranted. Both arms will complete a study-specific, day 14, in-person, follow-up review for verification of self-reports (intervention) and comparison (control). Data collection includes extraction of routine client MC records, study-specific database reports, and participant usability surveys. Intent-to-treat (ITT) analysis will be used to explore differences between groups to determine if two-way texting (2wT) can safely reduce MC follow-up visits, estimate the cost savings associated with 2wT over routine MC follow up, and assess the acceptability and feasibility of 2wT for scale up.

**Discussion:**

It is expected that this mobile health intervention will be as safe as routine care while providing distinct advantages in efficiency, costs, and reduced healthcare worker burden. The success of this intervention could lead to adaptation and adoption of this intervention at the national level, increasing the efficiency of MC scale up, and reducing burdens on providers and patients.

**Trial registration:**

ClinicalTrials.gov, NCT03119337. Registered on 18 April 2017.

**Electronic supplementary material:**

The online version of this article (10.1186/s13063-019-3470-9) contains supplementary material, which is available to authorized users.

## Background

Male circumcision (MC) reduces the risk of female-to-male HIV-1 transmission by up to 60% [1–3]. From 2008 to 2015, 10.4 million voluntary medical male circumcision (VMMC) procedures were performed [4], falling far short of the 20 million MC target [5] aimed at averting 3.4 million HIV infections through 2025 [6]. Although surgical VMMC has been streamlined [7], current VMMC follow up requires at least one in-person visit within 14 days of VMMC [8–10]. Severe shortages of healthcare workers [11] combined with rapidly expanding VMMC programs threaten already overburdened healthcare systems.

In pilot studies and controlled trials in sub-Saharan Africa, surgical adverse event (AE) rates from combined moderate and severe AEs range from 0.5% to 8% with few severe AEs resulting in permanent impairment [1–3, 12–19]. Although AE reporting in field settings is challenging [20–22], AE rates average 0.8% in southern African countries [23]. These low AE rates correspond to 99% of men healing without incident, leading to millions of healthcare visits conducted without cause and needlessly increasing the workload. For men, these unnecessary visits likely create barriers to care such as transportation costs, wait times, and inconvenience [24–28], potentially reducing VMMC uptake.

The spread of mobile telephones throughout sub-Saharan Africa affords an important opportunity to address these inefficiencies and retain quality care. Text messages show immense promise to positively influence health behavior and improve clinical care [29–31]; however, most short message service (SMS)-based health promotion efforts blast pre-defined messages to many people simultaneously [32–36], removing patients’ ability to communicate back with healthcare workers [37] and potentially reducing patient engagement in care. In contrast, two-way texting (2wT) between providers and patients shows promise to provide quality interactive care [38–41].

As a national VMMC implementing partner, the ZAZIC consortium, led by the University of Washington’s International Training and Education Center for Health (I-TECH) and local implementing partners, The University of Zimbabwe (UZ)-University of California San Francisco Collaborative Research Program, Zimbabwe Association of Church-related Hospitals (ZACH), and Zimbabwe Community Health Intervention Research Project (ZiCHIRe), works in partnership with the Zimbabwe Ministry of Health and Child Care (MoHCC) to implement VMMC services in 10 districts [42]. ZAZIC’s program is highly productive and safe: between July 2015 and June 2016, ZAZIC conducted 47,905 MCs and 45,989 follow-up visits and identified 151 AEs, giving an AE rate of 0.3% [43]. Adherence to follow-up visits, another indicator of program quality [44], was good. Over 88% of men reportedly attended both day-2 and day-7 follow up; more than 30% also had a day-42 visit to verify complete healing (P. Marongwe, personal communication, May 2016). With large numbers of follow-up visits, even if 20% of men were reviewed on days 2 and 7 for high sensitivity of AE detection, almost 74,000 in-person visits were unnecessary and could potentially be replaced with 2wT interactions between patients and providers. The aim of this study protocol, designed according to the Standard Protocol Items: Recommendations for Interventional Trials (SPIRIT) 2013 checklist (Additional file 1), was to implement a 2wT intervention that combines automated and interactive text messaging to maintain safety while dramatically reducing the burden that in-person VMMC follow up places on weak health systems.

### Trial design

A prospective, randomized controlled trial (RCT), our study compares two groups of clients with cell phones: (1) routine care (control group) and (2) clients who receive and respond to a daily text with in-person follow up only if desired or if an AE is suspected (intervention group). Both arms complete a study-specific, day-14, in-person, follow-up review for verification of self-reports (intervention) and comparison (control).

### Study objectives

#### Objective 1

Objective 1 is to determine if 2wT can safely reduce VMMC follow-up visits. We hypothesize that (1) 2wT is non-inferior to routine follow up for patient safety and that (2) 2wT will reduce unnecessary follow up compared to routine care.

#### Objective 2

Objective 2 is to estimate the cost savings associated with 2wT over routine VMMC follow up. We hypothesize that 2wT will reduce the costs associated with VMMC patient follow up compared to routine care.

#### Objective 3

Assess the acceptability and feasibility of 2wT for further scale up. We hypothesize that acceptability, usability, and feasibility will be high, aiding program scalability.

## Methods/design

### Study setting

2wT will be implemented in existing VMMC sites in the Chitungwiza District, a district purposefully selected for its high volume of VMMC clinic locations, and Norton Hospital, which is in Chegutu District. Chitungwiza District, a suburb of Harare, has an estimated 300,000 eligible, HIV-negative men ages 15–29 years; only 14% of eligible men have been circumcised to date. We will implement 2wT in up to five ZAZIC VMMC sites in Chitungwiza- Seke North clinic, Seke South clinic, Zengeza clinic, Chitungwiza central hospital and CitiMed hospital, sites averaging 50–200 VMMC/month. Norton Hospital performs on average 200–300 VMMC/month. All VMMC clients receive surgical MC as of March 2017, rather than PrePex device-based MC.

### Participant eligibility

VMMC client recruitment, voluntary consent (Additional file 2), and enrollment will be managed by the on-site 2wT study coordinator before VMMC surgery. Eligibility criteria for VMMC clients are (1) 18 years or older; (2) possesses own phone at enrollment; (3) provides contact details (phone, address, next of kin); (4) requests surgical VMMC; (5) is willing to follow MoHCC VMMC protocols; (6) is willing to come in on day 14; (7) is willing to respond to a questionnaire administered by phone 42 days after circumcision; and (8) experiences no intraoperative adverse event during routine VMMC. Men without cell phones and those who chose PrePex will be excluded as PrePex requires a device-removal visit 7 days after placement and has distinctly different follow-up protocols. As phone sharing practices are common, informed consent will ascertain whether eligible men have consistent access to a phone to receive messages about AEs. Participants who refuse enrollment will be asked to provide informed consent for data collection about reasons for non-participation. We expect to enroll and randomize 722 men in a 1:1 ratio into the intervention and control groups within 6 months, allowing observation of the primary outcome by the end of year 1. On day 14, all men in either arm who return for follow up will receive a USD$5 airtime credit. Men who have an inter-operative AE during routine VMMC will be withdrawn from the study as these men will have known, additional follow-up risks and mandatory in-person visits.

For healthcare workers (objective 3) within the six study sites, the inclusion criteria are as follows: employees posted at the site; at least 18 years of age or over; provide health care services to patients as part of the VMMC programs; and are able to provide written informed consent (Additional file 3). Healthcare workers will be excluded if they are not willing to participate or not willing to be recorded.

### Study participants

Study participants are described in Table 1. There will be 50 men enrolled in the pilot who will be enrolled in texting and not be randomized. These men will not be included in outcome analysis. There will be 722 men in the full study randomized in a 1:1 ratio of texting and control, with 361 men in the texting group and 361 in the control group (routine care).

**Table 1:** Study participants

Group name/description	Data collection method	Age range of subjects (years)	Target number of individuals
Pilot: assigned texting	Usability survey, routine VMMC clinical review; 2wT texting database	18–65	50
Main study: randomized texting	Usability survey, routine VMMC clinical review; 2wT texting database; day-14 review	18–65	361
	Subset of first 100 willing to complete the satisfaction and acceptability questionnaires		
	Time-motion subset		
Main study: randomized control group	Routine VMMC clinical review; day-14 review	18–65	361
Healthcare workers	Key informant interview	18–65	~ 8

*VMMC* voluntary medical male circumcision, *2wT* two-way texting

### Interventions

#### Technology development

**Fig. 1:**
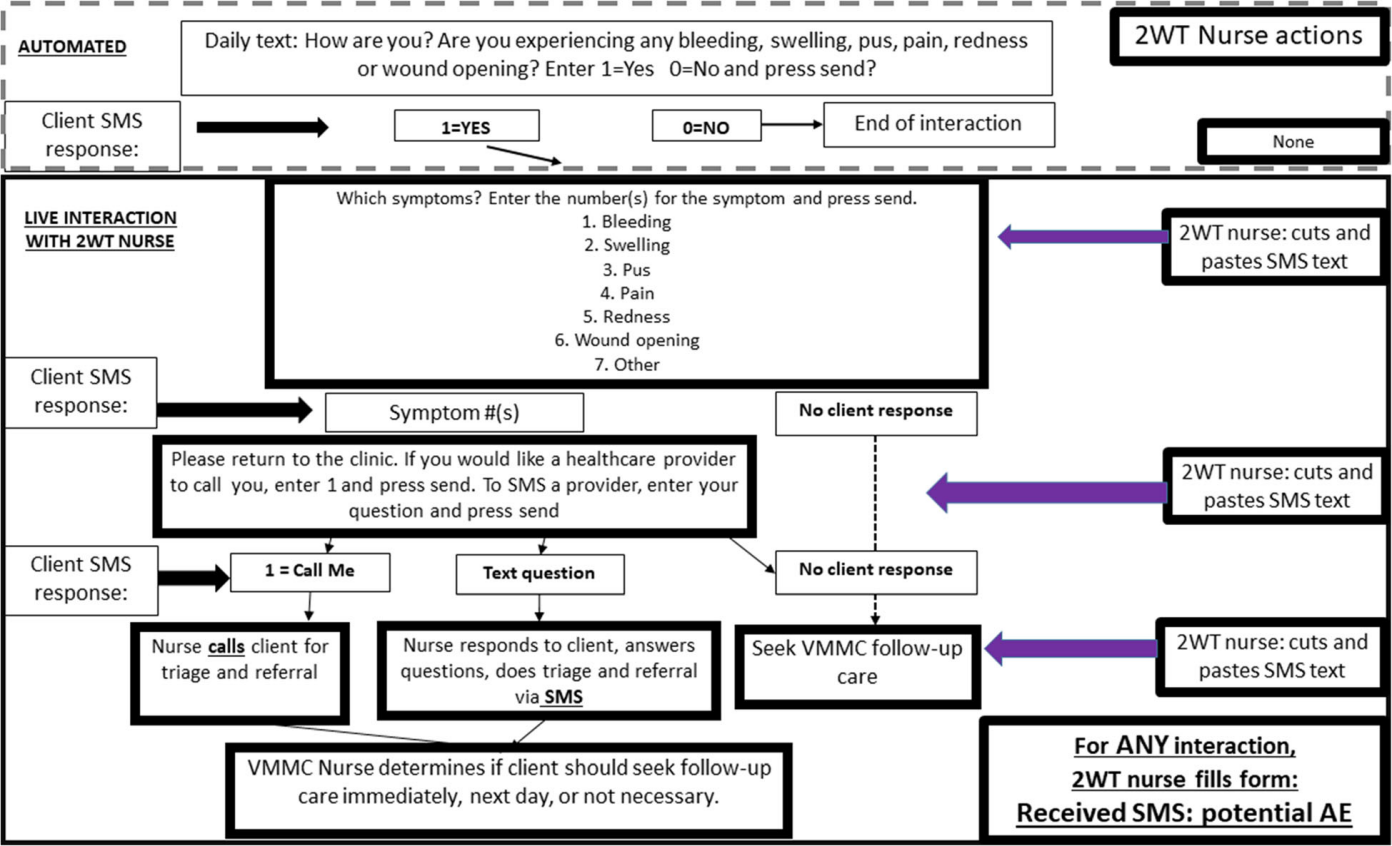
Two-way texting (2wT) nurse/patient interaction flow diagram. VMMC, voluntary medical male circumcision; AE, adverse event

Studies that demonstrate the impacts of mHealth interventions too often have evaluated technologies that are made “from scratch” and as a result are not robust enough to merit widespread replication [40, 45]. By partnering with a well-established non-profit mHealth organization, Medic Mobile (http://medicmobile.org/), and integrating with their existing software platform, open-source community and current efforts to integrate with existing health information systems (HIS) throughout sub-Saharan Africa, our team and proposed intervention are well-positioned to scale up and sustain any promising results. Since 2008, Medic Mobile has been a leader in the global mHealth community [46–48], equipping more than 13,500 health workers serving over 8 million people across 23 countries. The Mobile Medic Toolkit is an Android-based application that supports texting in any language and works with or without Internet connectivity on basic phones, smartphones, tablets, and computers [49]. The current, well-proven, app-based Toolkit that will be the basis for 2wT provides an automated and prioritized list of upcoming tasks, guiding health workers through actions (e.g., response waiting, referral). The Toolkit provides real-time progress indicators such as texting delivery rates and response rates. Data from mobile users are replicated to Medic Mobile web app and analytics tools for real-time response. The platform is highly configurable, currently supporting evidence-backed workflows and program implementation related to ensuring safe deliveries [50], tracking patients with tuberculosis [51], boosting immunization rates [52], and monitoring stocks of essential medicines [53]. These adaptable tools are free, open-source, and developed using human-centered design with input from people delivering care in the hardest-to-reach communities. While this technology has never been used in a VMMC program and requires adaptation of the clinical content, the existing 2wT software toolkit already contains the robust messaging features discussed in this proposal. The software adaptation process to alter and test alternative clinical content and the local specifications requires little additional software development. The adaptation process will be completed by collaborator, Holeman, with additional technical and training support provided by Nairobi-based technical experts. The automated versus manual texts are presented in Fig. 1.

#### Training of study personnel

VMMC staff at study sites will be informed of the study and briefed on study protocols. A 2wT study coordinator will be trained in confidentiality, protection of human subjects, enrollment, and data collection methods, working to not interfere with routine VMMC flow. For technology training, an experienced Medic Mobile trainer from Nairobi will work in person with the local study coordinator and team members to implement and learn to maintain the Medic Mobile toolkit independently. We believe that achieving local maintenance and ownership of the system is feasible because of Medic Mobile’s considerable investments in open-source collaboration and in designing software that is user friendly and intuitive enough to be adapted and maintained by local staff. As part of 2wT, one experienced VMMC nurse will serve as Study Coordinator in Harare and will manage the texting database, including text and voice follow-up communication with intervention clients.

#### Study pilot

We will conduct a rapid situation analysis with healthcare workers, VMMC clients, and stakeholders to assess suitable responses to VMMC client texts, setting standards for text responses and in-person follow up. We will modify existing usability surveys for this public health context. A small pilot with 50 VMMC clients who will be enrolled in the texting intervention will include usability testing with both 2wT clients and nurses implementing the 2wT system, illuminating system experiences from both perspectives. Usability results will inform in-box modification, message format preferences (SMS or WhatsApp) and optimal message delivery (timing, frequency, language preferences). Experience from the study coordinator and other team members will add detail to inform 2wT adaptation and modification of standard operating procedures before implementation. We will examine local infrastructure (e.g., electricity and cell network), and explore 2wT cost reduction options (e.g., text bundling, free call-back numbers) and adapt accordingly.

#### Routine VMMC care (control arm)

For the 361 men randomized into the control arm, ZAZIC follows all MoHCC protocols based on World Health Organization (WHO) guidelines [8] including routine surgical VMMC follow up on post-surgery days 2, 7, and 42 (Table 2). Patients may seek care for suspected AEs outside scheduled visits, at any healthcare facility at any time, but most often return to their VMMC site. Referral cards for VMMC clients provide local numbers for patients to text, call, or request a call-back for emergencies. A standardized approach is used to assess, identify, and record the severity of AEs [44]. All VMMC care, from assessment of AEs through complete healing, is provided free to clients from MoHCC. Clients who do not return to the clinic for follow up on day 2 or day 7 are traced: three attempts by phone and then up to three attempts in person are made to track the clients, after which they are considered lost to follow up (LTFU) (P. Marongwe, personal communication, May 2016). Clients are not traced for day-42 visits. For the purposes of this study, control-arm VMMC clients will be asked to come in on day 14 for an additional follow-up visit. No active follow up is provided at day 14 for routine clients who miss the study-specific day 14 visit.

**Table 2:** Procedures for intervention and control

	Routine (Control)	Intervention (2wT)
Day-0 routine VMMC registration and client intake forms	X	X
Day-0 VMMC surgery and counseling	X	X
Day-0 study consent	X	X
In-person follow up		
Routine day 2	X	
Routine day 7	X	
Study-specific day 14	X	X
Routine day 42	X	
Routine lost-to-follow-up tracing		
Day 2	X	X
Day 7	X	X
Daily texts days 1–13		X
MoHCC routine AE procedures		
In-person, any day, follow up for suspicion of AE	X	X
Emergency VMMC after-hours care	X	X
AE identification	X	X
AE severity grading	X	X
AE management and treatment	X	X
AE reporting on routine MoHCC forms	X	X

*VMMC* voluntary medical male circumcision, *2wT* two-way texting, *MoHCC* Zimbabwe Ministry of Health and Child Care, *AE* adverse event

#### VMMC care procedures (intervention (2wT) arm)

We will conduct a prospective, un-blinded RCT among VMMC clients in a 1:1 ratio of control to intervention. Study participants and clinic staff are not masked to treatment. The 361 men randomized to the intervention arm (2wT) will receive routine VMMC surgical care and counseling, including referral cards for emergencies. The 2wT clients will receive automated daily texts from day 1 to day 13 (Table 2). Texting language may be in English, Shona, or Ndebele at the choice of the participant. It is free to receive call and texts; it costs USD $0.05 to send an SMS in Zimbabwe [54]. If they respond that they suspect no AE, no immediate follow-up action will be taken. If a 2wT VMMC client responds affirmatively to any daily text that he suspects an AE, a VMMC nurse will exchange modifiable, scripted texts with the client to determine the symptoms, frequency, and severity. Then, if deemed necessary, the client will be asked to return to clinic the following day or earlier if an emergency is suspected. AEs will be managed in adherence to MoHCC routine care. If 2wT clients do not respond to texts on day 2 or day 7, the same MoHCC tracing process will be activated, after which they will be considered LTFU. All study participants will be asked to come to the clinic for study-specific, day-14 follow up to review healing and verify AE reporting. Day 14 was chosen for verification because 95% of all AEs within the ZAZIC VMMC program are reported on day 14 or earlier [55], suggesting that most AEs have occurred by this time point. In a previous field study of AEs, the most common AEs of bleeding and infection were identified a mean of 6.7 and 9.0 days after VMMC, respectively [20], further supporting the 14-day period used in this and a previous study [13]. The day-14 review will be conducted by routine VMMC providers according to MoHCC review guidelines. At day 42, we will implement a brief text-based survey with 2wT clients to ascertain complete healing, providing stronger inferences at study completion. Intervention-arm clients could withdraw at any time for any reason and continue with routine VMMC care provision.

No compensation will be provided for healthcare workers. A USD$5 cell phone credit will be given to both control and intervention arm participants on day 14 in appreciation of their time.

### Outcomes

**Fig. 2:**
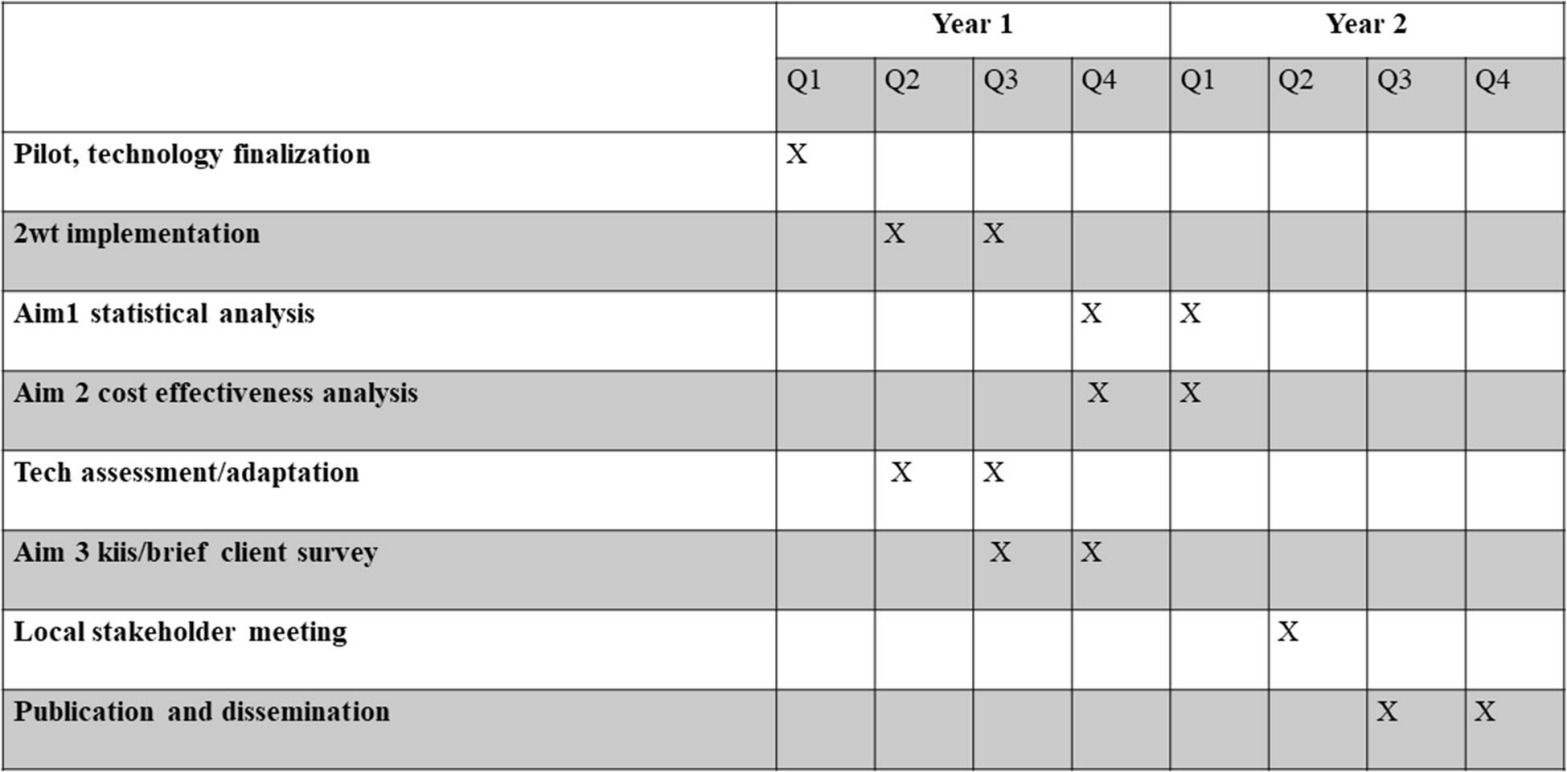
Study implementation timeline. 2wT, two-way texting; KIIs, key informant interviews

For objective 1, the primary safety outcome is rate of combined moderate or severe AEs ≤ 14 days post VMMC. The workload outcome is the average number of in-person follow-up visits in the control compared to the intervention arm at day 14. For objective 2, the outcome is an estimate of the incremental intervention costs relative to routine practice to quantify gains in healthcare efficiency for scale up and adoption. For objective 3, the outcome is an assessment of the feasibility, acceptability, and usability of 2wT for replication and sustainability in Zimbabwe and the region. The intended participant timeline is presented in Fig. 2.

### Sample size

For objective 1, we will apply hypothesis testing for non-inferiority to examine the outcome of interest: AE rate (moderate or severe) occurring ≤ 14 days. A non-inferiority trial is appropriate as we wish to test our assumption that 2wT with reduced in-person follow up is as safe as (not clinically nor statistically inferior to) routine VMMC [56, 57], but with additional advantages including efficiency and lower direct and indirect costs to providers and patients. The non-inferiority margin, based on statistical and clinical considerations, is the maximum difference between the rate of AEs ≤ 14 days in the control and 2wT arms where we would conclude that 2wT is not inferior to routine care. We set that margin to 1.6%, which would create a non-inferiority cutoff of ≤ 2% AEs in 2wT. The cutoff for the proportion of AEs to define 2wT non-inferiority was set at a conservative 2% of AEs to match the lower bound of AE rates reported in previous rigorous studies [1–3] and because an AE rate of 2% is regarded as a commonly used standard of VMMC safety [58, 59], including in Zimbabwe [60]. Including a possible 10% LTFU, 361 participants per arm provides 90% power at alpha of 2.5% to detect AEs above the non-inferiority margin of 1.6% (Table 3). We would conclude that 2wT is not inferior if we can rule out an AE rate > 2%.

**Table 3:** Sample sizes at different non-inferiority margins^a^

Non-inferiority margin	AE proportion cutoff for 2wT non-inferiority	Sample size (number per arm)
0.6%	1% AEs or less	2326
1.6%	2% AEs or less	328
2.6%	3% AEs or less	124

*2wT* two-way texting, *AE* adverse event

^a^Using ssi command from STATA 12.0 for one-sided, non-inferiority

### Recruitment

The City Health Director who manages the national VMMC program at the Provincial level in conjunction with our implementing partners will facilitate access to the clinics/hospitals where ZAZIC provides ongoing support for VMMC. We will also seek permission from the facility manager to conduct the study at that particular clinic. It would be expected that demand would be created in anticipation of the study implementation.

Each site will establish local recruitment and screening methods that operationalize protocol-specified requirements for determination of eligibility in a manner that is tailored to and most efficient for the local study setting and target study population. In brief, information about the study will be disseminated through the MoHCC, ZAZIC, and healthcare workers (HCW) at the selected sites. The creation of demand for VMMC as part of routine ZAZIC program practice will support study recruitment. HCW recruited for participation in the study will be reached at their workplaces (the healthcare facilities) following communication between the study team and the site leadership. VMMC clients will be recruited in the VMMC clinic area. Recruitment will be managed by specifically trained research coordinators who receive payment for their roles as part of routine or study work. The research coordinators will meet with VMMC clients to sensitize them to the opportunity to participate in a study of text-based follow up; those meeting eligibility criteria will be individually informed by 2wT staff about the opportunity to participate in the study. Interested men will be referred to the site study coordinator for study enrollment and informed consent. This person will meet with the men in a private setting, further explain the study, confirm study eligibility, and seek informed consent.

### Randomization process

We will create and allocate 361 “texting” and 361 “routine” (ensuring 10 extra per group in case of withdrawals) envelopes. Envelopes are block-randomized in groups of 20 using manual shuffling to ensure a near-random order. The full set of 722 will be numbered before distribution to sites. A set of 20 extra envelopes will be created - 10 per arm - to allow replacement of withdrawals. Each group-assignment envelope will be drawn by the site coordinator and then opened with the participant. Security envelopes will be used to help prevent selection of assignment to a group. Subjects within each block are randomly assigned to treatment conditions.

### Data collection, management, and analysis

During informed consent, participants will be asked to give permission to use data from their routine VMMC medical records, including the VMMC register and client intake form (CIF). Data from paper CIFs includes VMMC number, age, circumcision type, eligibility criteria, pre-procedure assessment, and AEs during the procedure. Variables of interest from CIFs and the register will also be entered using Medic Mobile software and verified through data checks, easing merging with the SMS database data management. De-identified, coded data will be shared between researchers via secure networks.

For objective 1, the 2wT safety outcome of interest is the cumulative rate of AEs (moderate or severe) ≤ 14 days. The incidence of AEs before day 14 will be extracted from routine VMMC data for both 2wT and control. Incident AEs on day 14 will be identified, classified, and graded for severity using routine MoHCC protocols [9] and recorded on routine VMMC AE forms. We will compare cumulative rates of any moderate or severe AEs ≤ 14 days in the two groups using Fisher’s exact test, as the expected number of AEs is small. The rates will be calculated per arm as: (# moderate + severe AEs)/(total # VMMC clients who attend/responded to 2-day, 7-day, or 14-day follow up visits or texts). Multivariate logistic regression models (any AE versus none) will be used to quantify the magnitude of difference, adjusting for any potential confounders. To determine the reduction in follow-up visits, we will compare the mean number of in-person visits for intervention and control by *t* test. A multivariate linear regression model will be used to further quantify the effect of intervention on reduction in the number of visits, adjusting for potential confounders. Secondary outcomes include AE rates on day 14, texting response rate, time between 2wT AE text reporting and follow up, and severity of AEs. We will perform appropriate summaries of missing data and LTFU, testing for systematic differences in response by study arm. Data will be exported from the study database into STATA 12.0 for analysis.

For objective 2 (2wT costs), we will calculate the relative costs and outcomes (effects) of intervention versus control, including costs for technology, healthcare worker time, and client considerations (travel, text costs, missed work). We will conduct both activity-based costing from the implementation perspective and from the technology perspective to extrapolate our results as costs that would be incurred by the MoHCC should they elect widespread scale up of 2wT. The approach used for costing is as follows. First we quantify the total direct and indirect costs of 2wT deployment, a method previously used for technology cost assessment in Malawi [61], which includes comprehensive costs from installation and training, routine maintenance, healthcare worker time associated with 2wT, and staff efficiency gains/losses in addition to the direct cost of technology. Second, we will estimate incremental costs (incremental relative to routine practice) for the intervention. This component entails a micro-costing study using activity-based approaches for costs incurred (training, VMMC service provision, follow up) and costs averted (health costs for providers and patients saved by reducing the number of visits), adapting previous VMMC costing estimates [62] for 2017 Zimbabwe dollars when appropriate. Cost data will also be collected from the study budget, public health clinic budgets, published government reports, and the health economics literature. Last, to estimate the cost savings associated with 2wT over routine VMMC follow up, we will conduct a time-motion study [63, 64] to quantify time spent for VMMC follow up, indicating potential time savings for providers and VMMC clients. One trained observer will record VMMC client/provider follow-up interactions for 5 days, recording times and activities using a pre-established checklist and a personal digital assistant (PDA) that assigns start and end times. During these same 5 days, client time from registration through visit completion will be recorded by a second observer. Data will be exported into STATA 12.0 for analysis. The combined, overall costs for the delivery of 2wT in public health clinics will be estimated and compared to routine follow up.

For objective 3 (2wT acceptability), using study recruitment and enrollment logs in addition to the texting database, we will describe levels of acceptance, participation, refusal, and drop-out. We will carry out key informant interviews (KIIs) with up to eight healthcare workers to gauge acceptability and satisfaction, identify facilitators and barriers to program success, and ascertain suggestions for intervention improvement. KIIs will be audio recorded and transcribed. The Atlas.ti software will be used to create a spreadsheet of key themes, perceived barriers, and suggested facilitators to the program from KIIs. We will also implement questionnaires at the day-14 visit with a subset of 100 2wT VMMC clients in the main study who were randomized to texting, to gauge satisfaction, estimate direct and indirect costs (time away from work, transportation costs), and ascertain suggestions for intervention improvement. Responses from the brief, self-administered, quantitative surveys with VMMC clients will be entered into Microsoft Excel and frequencies explored in STATA 12.0. For feasibility, costing data will be combined with usability and acceptability information. Qualitative data will be entered, coded and analyzed as text documents in Atlas.ti 6. The first-line data quality assurance will be the responsibility of the I-TECH investigators. These files will also be sent to the team in Seattle, USA for secondary data quality assurance and analysis. The recordings of the interviews will be destroyed 1 year after the activity ends. However, the transcriptions will be kept for 5 years in compliance with University of Washington policy. These comprehensive data will be discussed at a final stakeholder meeting to disseminate study results, validate interpretations, and refine recommendations for future scale up and modification of 2wT in Zimbabwe and beyond. Additional meetings between local researchers and the MoHCC will further determine feasibility for replication and scale up.

### Intervention monitoring

We will form an independent Data Safety and Monitoring Board (DSMB). The DSMB will be available to review and advise on any concerning findings, such as social harms or other AEs linked to the study, distress evoked among participants that might outweigh the potential benefits of the study, difficulties in recruitment, difficulties in study site participation, or any other issues that threaten the scientific or ethical foundation of the study. The DSMB will have the power to stop the study at any time. The investigators will also reach out to the University of Washington (UW) Institutional Review Board (IRB) and members of the Medical Research Council of Zimbabwe (MRCZ) in the event of concerns related to participant well-being or study validity, to consult on whether study procedures need to be modified or halted.

### Ethics

This study and interim modifications were approved by both the MRCZ and the UW IRB. No deviations from the protocol after approval will be implemented without prior approved amendment by these IRBs except where it may be necessary to eliminate any immediate hazard to study participants. In such a case, the deviation will be reported to the IRBs as soon as possible. Written informed consent will be obtained from all participants prior to enrollment in the study.

### Dissemination

The results of this study will be presented at specific scientific conferences in Zimbabwe and will be submitted for publication in peer-reviewed journals.

## Discussion

This study is innovative in several ways. First, we aim to replace in-person follow-up visits with 2wT interaction between clients and providers, which holds potential to dramatically reduce the burden on the health system at low cost while maintaining patient safety. This rigorous research involving local researchers is needed to test and optimize the approach. Moreover, we aim to implement 2wT within an existing MoHCC VMMC program structure that will greatly improve the likelihood of program scalability and sustainability. We will also quantify expected MoHCC cost savings provided by 2wT scale up. This study sets a precedent by employing digital technology to identify cases of uncomplicated healing and thereby to reduce the burden of unnecessary VMMC clinic visits for both providers and clients - thus opening up the use of mHealth in post-operative care or other contexts with low rates of complications, potentially even long-term use of antiretroviral therapy. Last, as the rate of unnecessary, in-person care in VMMC programs is likely high, potential gains in efficiency identified through this low-cost digital innovation are large, increasing the likelihood of scale up and replication in the region.

Although it is expected that this trial will yield important data to inform VMMC program expansion in Zimbabwe, there are some limitations to the potential conclusions and generalizability of the findings. Men undergoing PrePex VMMC are excluded, limiting generalizability to surgical VMMC. Phone ownership in Zimbabwe’s urban areas is high, but there is a possibility that men who undergo VMMC have lower-than-estimated phone access, reducing the potential efficiency of a texting approach to follow up. While evidence from Kenya and elsewhere suggests that patients are likely to interact with health professionals via SMS, it is possible that a higher-than-estimated percentage of clients will not respond to SMS, increasing the need for follow up by voice calls, thus reducing the efficiency and cost gains afforded by the intervention. We anticipate some challenges in program implementation such as network outages (though these are increasingly fewer and shorter) and user error, but previous Medic Mobile experience and the expert local team will enable swift identification of bottlenecks and appropriate, real-time solutions.

If texting VMMC clients is found to be non-inferior to routine, in-person follow up for detection of AEs, scale up could dramatically reduce time spent on unnecessary VMMC follow-up visits by healthcare personnel and decrease the burden on VMMC clients. Future funding could enable scale up of the intervention in other high-volume VMMC sites with high mobile phone coverage, conducting additional rigorous evaluation in diverse field and clinical contexts. We would seek funding to test 2wT among rural populations or smaller urban areas. Last, we will explore how 2wT could be integrated into DHIS2, the national health information system, to improve and ease reporting.

## Trial status

Recruitment of participants into the pilot phase of the trial ran from 16 April 2018 to 4 May 2018. For the full study, recruitment in one of two study sites (Seke South) began on 20 June 2018 and in the other site (Norton) on 11 December 2018. Recruitment was completed on 11 February 2019.

## Additional files

Additional file 1: SPIRIT checklist. (DOC 121 kb)

Additional file 2: Material consent form - MC clients. (DOCX 537 kb)

Additional file 3: Material consent form - clinicians. (DOCX 34 kb)

## Data Availability

The final (anonymized) trial dataset will be accessible for investigators for final analyses. The datasets may be made available to external researchers upon request and approval of a data sharing agreement.

## Abbreviations

2wT: Two-way texting
AE: Adverse event
CIF: Client intake form
HIV: Human immunodeficiency virus
IRB: Institutional review board
I-TECH: International Training and Education Center for Health
KII: Key informant interview
LTFU: Lost to follow up
MC: Male circumcision
MoHCC: Zimbabwe Ministry of Health and Child Care
SMS: Short message service
SPIRIT: Standard Protocol Items: Recommendations for Interventional Trials
UW: University of Washington
VMMC: Voluntary medical male circumcision
