# Supplementary material for: Reducing provider workload while preserving patient safety via a two-way texting intervention in Zimbabwe’s voluntary medical male circumcision program: study protocol for an un-blinded, prospective, non-inferiority, randomized controlled trial
Source: Trials. 2019 Jul 23;20:451. doi: 10.1186/s13063-019-3470-9 (PMC6651991; doi:10.1186/s13063-019-3470-9)
Supplement: Supplementary file 2 — Material consent form - MC clients. (DOCX 537 kb) [file 13063_2019_3470_MOESM2_ESM.docx]

Appendix 2: Informed consent form for men

MRCZ PROTOCOL #_______________

#

# INFORMED CONSENT FORM

CLIENT CODE_________________

**Reducing provider workload while preserving patient safety: a 2-way texting intervention in Zimbabwe’s voluntary medical male circumcision program**

**Principal Investigator:** [removed]

**Phone numbers:** [removed]

**Study sites:** Zengeza Clinic, Seke South Clinic, Seke North Clinic, CitiMed Hospital, Chitungwiza Central Hospital and Norton Hospital.

**What you should know about this research study:**

- We give you this consent so that you may read about the purpose, risks, and benefits of this research study.
- The main goal of research studies is to gain knowledge that may help people in the future.
- We cannot promise that this research will benefit you.
- Your participation is voluntary. You have the right to refuse to take part, or agree to take part now and change your mind later.
- You should only volunteer for the study if you are willing to use your own cell phone to communicate daily with the study team about your post circumcision healing
- You should only volunteer for the study if you are willing to come in on Day 14 for in-person follow-up
- The study will compare two different ways to provide follow-up care and information to men who received circumcision. Half of those who enrol in the study will undergo routine, in-person care while the other half have text-based follow-up. You will be randomized to the texting follow-up or not.
- Please review this consent form carefully. Ask any questions before you make a decision.
- If you need urgent medical assistance related to the circumcision there is a staffed, 24 hour number to provide assistance. This number is: XXXXXXXXX

# PURPOSE

You are being asked to be part of a research study to learn whether text-based follow-up after circumcision is as safe as in-person follow-up to ensure proper wound healing. In this study, there is an option of no mandatory visits after MC. Men will text with the study nurse daily for 14 days. Men come in only if they are concerned about their healing. Most men heal without any complications. What we learn from this study will help the Ministry of Health and Child Care (MOHCC) decide if men, themselves, can determine if they need to be seen by a clinician after circumcision. If most men can safely be followed-up by texting, and only those concerned about their healing come in for in-person review, it would save both time and money for men and healthcare providers.

The study will enrol men from Chitungwiza and nearby areas. The first 50 men will participate in the pilot study and will receive the texting follow-up reviews. The 722 men in the actual study will be randomized: half of those will be in the text-based follow-up after surgical male circumcision (MC), and half will have routine follow-up. We will also help determine how long follow-up reviews take in the clinic.

You were asked to consider being part of this study because the opinions and experiences of men like you affect the national MC program. We will be able to find better ways to follow-up with men and ensure safe post MC healing using the information you provide in this study, including your experience with taking care of the wound and your thoughts on the texting follow-up process. This study will also allow the MOHCC to learn the best way to offer MC to the whole country.

Of course, you may select to not take part in this study. Your participation in the study or your decline to participate will not affect your care.

# PROCEDURES AND DURATION

Screening Procedures:

If you decide to be part of the study, and you are eligible and consent for surgical MC according to the MoHCC guidelines, here is what will happen before you are circumcised. We will meet you in a private place, and a trained nurse will briefly explain the study, and ask if you agree to be part of the study. If you agree, we will record your name and ask for your consent to use your routine MC records, including demographic and clinical information as used in routine care. We will ask you a few additional questions about your wages and transportation costs. We will also check your phone to make sure it will work for the study purposes. You will need to ensure you have enough cell credit to respond to daily texts. You will receive $5 in cell phone credit on Day 14.

If you choose to take part in the study, you will be "randomized" into one of the two study groups described below. Randomization means that you are put into a group by chance. A number in an envelope will place you in one of the study groups. Neither you nor your nurse can choose the group you will be in. After the first 50 who will receive text-based follow-up as part of the pilot, you will have an equal chance of being placed in either group. Group 1 is the texting group. Group 2 is the routine care group. This number and assignment cannot change.

.

Study Procedures: Routine care group

If the randomization tell us that you are placed in the routine care group, you will undergo VMMC and follow-up as per routine care including in-person follow up on Day 2 and Day 7. As part of the study, you will be required to return to the clinic for an extra visit on Day 14 for clinical review. You may be observed during a follow-up visit to determine your time spent in the clinic as part of the study. On Day 14, you will receive $5 in cell phone credit. You will then be done with the study, but will be told to return for any unexpected problem with your circumcision and cared for by the clinic team as per routine care.

Study Procedures: Texting group

If the randomization tells us that you are placed in the study intervention group for text-based follow-up, you will be asked to give your phone numbers and agree to be texted, and reply by text, every day for 14 days. You will be followed-up by home visit if you miss your daily text. You are reminded that you may seek in-person follow-up if you want at any time, just as with routine care. You will also have access to the emergency services as with all MC clients.

Before the actual circumcision procedure, we will review home-based wound care, just as we would for routine care. We will then show you how to remove your own bandage on Day 2, which you will do at home. We will show you photos of what normal healing looks like and how to identify any concerns in healing. We will answer any questions you have.

We will review the daily texting process, show you what the texts will look like, and review your possible responses. We will also explain how you can text to a study nurse at any time if you have concerns. We will also explain how you can have a nurse call you if you have any concerns.

As part of this study, there would be no mandatory post-MC visits. But as with routine follow-up, men may come for in-person review at any time if you have any concerns at all. You will have a surgical MC as with routine care. You will consent to surgical MC separately from this study consent.

Before your MC, we will send you an example text and make sure you can receive and respond on your phone. Each day after MC, we will send you a message that you need to respond to. On Day two, we will send you a reminder text to remove the bandage as well as instructions about bandage removal. Then, we will text you about your bandage removal experience. Then, we will text you every day until day 14. A response is required. Then, on Day 14, you will come for an in-person clinical review. You will receive your $5 phone credit then. The first 50 men (those in the pilot phase) will also be asked some brief questions about the actual text messages, including their length and content, to help make the study better. 100 men in the full study will be asked to answer some written questions about your experiences on Day 14 as well. In a chosen week, some of you will be observed moving through the clinic during a review visit to see how long your visit lasts. You will also receive a brief survey on Day 42 which will be completed by text to follow-up on your healing progress and satisfaction. You will then be done with the study, but will be told to return for any unexpected problem with your circumcision. If complications or delayed healing require longer follow-up, you will be followed by study staff as long as necessary or until study close-out. If clinical care is required after study close-out, care will be provided at the study site as part of regular clinical service. If necessary, you may be referred to a hospital near the study site for additional care.

**RISKS AND DISCOMFORTS.**

For men in the routine care group, there are no additional risks other than the need to return for the Day 14 visit.

For men in the text-based follow-up group, any additional MC-related risk that may be incurred by participants by virtue of their text-based follow-up will be monitored closely. Although there are no routine follow-up visits at 2 and 7 days, you will be communicated with by daily texts to check on your healing. You are reminded to seek in-person follow-up if you suspect an adverse event (AE) or if you desire in-person follow-up for any reason. You will receive referral cards for after hours and emergency care as those with routine care, and you are encouraged to use them if you need after hours or emergency care. The availability of staff in person or phone is same as for the routine care arm. Overall, the type of the follow-up is changing but not frequency or access. AE management will be conducted according to MoHCC guidelines as with all MC clients. Also, your recent MC may be known to others outside the study as a result of the texting intervention if others view your cell phone. You may also feel uncomfortable answering questions about your wages, employment, transport costs, service quality and your opinions about the intervention; however, we hope we will reduce these risks as much as possible. You can leave the study at any time with no repercussions.

# ALTERNATIVE TO TAKING PART IN THIS STUDY

# If you are not interested in participating in this study, you may have routine follow-up after surgical male circumcision as per standard care at this same site.

# BENEFITS and REIMBURSEMENT

We cannot promise that you will receive any benefits from this study. However, future clients could save time and money by only attending the reviews that were desired and forgoing compulsory reviews. This would ensure their safety and peace of mind while avoiding unnecessary follow-up. The research will also help Ministry of Health and Child Care (MOHCC) to make male circumcision more accessible and reduce costs incurred by men who desire VMMC.

You will be given $5 airtime credit at the Day 14 visit in appreciation for participating in the study.

# CONFIDENTIALITY

The information you give us will be kept private. No one outside of the study will know you participated. Any information that could be used to identify you will not be used in any reports or publications from this study. The investigator will keep information connecting your name to your results according to the retention period required by the University of Washington in accordance with state and/or federal law. This list will be kept locked in a separate file cabinet that only the study manager can access.

MRCZ, RCZ, US Government or University of Washington staff sometimes review studies such as this one to make sure they are being done safely and legally. If a review of this study takes place, your records may be examined. The reviewers will protect your privacy. The study records will not be used to put you at legal risk of harm.

# RESEARCH-RELATED INJURY

# If you experience any problems related to the study, you should call the study coordinator at

# XXXXXXXXXX or return to this clinic or any local clinic. If you experience any complication or injury as a result of the study, care will be provided at no cost to you.

# VOLUNTARY PARTICIPATION

It is up to you whether you want to be part of this study. If you decide to be in it, you may stop at any time. These decisions will not affect your future medical care from the MOHCC or partners. If you decide to leave the study, we will ask you for information about why you are choosing to leave. It is up to you whether to answer these questions.

**What if you have questions about this study?**
You have the right to ask and receive answers to questions about this research. If you have questions, complaints, or concerns, contact the researchers listed below:

1. ***[removed]***

# SIGNATURE PAGE

**Reducing provider workload while preserving patient safety: a 2-way texting intervention in Zimbabwe’s voluntary medical male circumcision program**

# Version 2 August 1, 2018

# OFFER TO ANSWER QUESTIONS

Before you sign this form, please ask any questions on any part of the study that is not clear to you. You may take as much time as you need to think about it.

# AUTHORISATION

I am making a decision about whether or not to participate in this study. My signature indicates that I have read and understood the information provided above, have had all my questions answered, and I have decided to participate.

____________________________________ __________

Name of Research Participant (please print) Date

__________________________________ Time in 24:00 format ___________

Signature of Participant

________________________ ______________________

Names of Study Staff Signature of Staff Obtaining Consent

Name of Witness Signature of witness date/time

**YOU WILL BE OFFERED A COPY OF THIS CONSENT FORM TO KEEP.**

If you have any questions concerning this study or consent form beyond those answered by the investigator, including questions about the research, your rights as a research participant or research -related injuries; or if you feel that you have been treated unfairly and would like to talk to someone other than a member of the research team, please feel free to contact the Medical Research Council of Zimbabwe (MRCZ) on telephone XXXXXXXXXX and cell phone lines XXXXXXXXX. The MRCZ Offices are located at the National Institute of Health Research premises at Corner Josiah Tongogara and Mazowe Avenue in Harare.
