# Supplementary material for: Reducing provider workload while preserving patient safety via a two-way texting intervention in Zimbabwe’s voluntary medical male circumcision program: study protocol for an un-blinded, prospective, non-inferiority, randomized controlled trial
Source: Trials. 2019 Jul 23;20:451. doi: 10.1186/s13063-019-3470-9 (PMC6651991; doi:10.1186/s13063-019-3470-9)
Supplement: Supplementary file 3 — Material consent form - clinicians. (DOCX 34 kb) [file 13063_2019_3470_MOESM3_ESM.docx]

MRCZ PROTOCOL #_______________

## **INFORMED CONSENT FORM**

MOHCC – 2WT STUDY

MINISTRY OF HEALTH AND CHILD CARE

UZ-DCM- ZiCHIRe Office: 28 Van Praagh, Harare, Tel: 791649

2-way texting intervention in Zimbabwe’s voluntary medical male circumcision program

Principal Investigator : [removed]

**Funding Source and/or Sponsor: National Institutes of Health, USA**
 
**Study Contact telephone numbers:**

| Name | Position | Phone number |
| --- | --- | --- |
| **24 Hour Urgent Assistance** |  |  |
| [XXXX] | Study Coordinator | [XXXX] |
|  |  |  |

## **INFORMED CONSENT FORM**

**What you should know about this research study:**

- We give you this consent so that you may read about the purpose, risks, and benefits of this research study.
- The main goal of research studies is to gain knowledge that may help people in the future.
- We cannot promise that this research will benefit you.
- We want to document your opinions about, and satisfaction with, the 2-way texting (2WT texting) follow-up study
- You have the right to refuse to take part, or agree to take part now and change your mind later.
- Please review this consent form carefully. Ask any questions before you make a decision.
- Your participation is voluntary.

# PURPOSE

You are being asked to be part of a research study to learn whether text-based follow-up after circumcision is as safe as in-person follow-up to ensure proper wound healing. In this study, there are no mandatory visits after MC. Men will text with the study nurse daily for 14 days. Men come in only if they are concerned about their healing. Most men heal without any complications. What we learn from this study will help the Ministry of Health and Child Care (MOHCC) decide if men, themselves, can determine if they need to be seen by a clinician after circumcision. If most men can safely be followed-up by texting, and only those concerned about their healing come in for in-person review, it would save both time and money for men and healthcare providers.

The study will have 772 men from Chitungwiza and nearby areas who agree to have text-based follow-up post surgical male circumcision (MC). It will also include up to 8 clinicians at study sites will also be asked questions about this study and their thoughts on text-based follow-up. As a clinician, you be asked questions such as what you know and think about the texting study. Some clinicians will also be asked about the formation of the messages and the SMS system as part of an SMS input session as part of the pilot of this study.

You were asked to consider being part of this study because the opinions health care providers affect many men’s decisions about MC. We will be able to find better ways to encourage more men to get MC using the information you provide in this study, including how satisfied you are with the texting intervention, your experience with taking care of these study participants and any problems caused by the follow-up intervention. We will also be able to improve the intervention with your input.

# PROCEDURES AND DURATION

If you decide to participate the following procedures will happen: We will seek to meet you in a private place and seek informed consent from you. As part of the initial pilot study, some of you may take part in initial conversations about the SMS system and its use from the clinician perspective. Any observations and discussions about the formation of the SMS and the messaging system from the SMS input session will take under 1 hour. For the main intervention, you will go through a one-to-one interview using a structured questionnaire after the intervention has been implemented. The interview will take place in a private space within the clinic and will solicit your opinions and experiences regarding deploying 2WT among men who come in for a circumcision procedure. We will ask you questions like, “What were the challenges of the texting system?” The interview will take approximately 30 minutes. With your consent, the interview will be recorded.

**RISKS AND DISCOMFORTS**

Taking part in the study may cause some psychological discomfort because you will be asked to provide your opinions regarding text-based follow-up after surgical circumcision.

**BENEFITS and** **COMPENSATION**

We cannot promise that you will receive any benefits from this study. The research will help Ministry of Health and Child Care (MOHCC) to make male circumcision more available and keep it safe. No compensation will be offered.

# CONFIDENTIALITY

The information you give us will be kept private. All information we collected during interviews will be locked away or kept on protected computers. No one outside of the study partner will know the results of the SMS input session or your interview. Any information that could be used to identify you will be shared only with your permission, and will not be used in any reports from this study. The recordings of the interviews will be destroyed one year after the activity ends. However, the link between your identifier and the research data will be destroyed after the records retention period required by the University of Washington in accordance with state and/or federal law. These records will be kept locked in a separate file cabinet that only study staff can enter.

MRCZ, RCZ, US Government or University of Washington staff sometimes review studies such as this one to make sure they are being done safely and legally. If a review of this study takes place, your records may be examined. The reviewers will protect your privacy. The study records will not be used to put you at legal risk of harm.

# VOLUNTARY PARTICIPATION

It is up to you whether you want to be part of this study. Your alternative is to not participate in the study. If you decide to be in it, you may stop at any time. These decisions will not affect your employment or future relationship with the MOHCC or its partners. If you decide to leave the study, we will ask you for information about why you are choosing to leave. It is up to you whether to answer these questions.

**What if you have questions about this study?**
You have the right to ask and receive answers to questions about this research. If you have questions, complaints, or concerns, contact the researchers listed below:

1. ***[removed]***

# OFFER TO ANSWER QUESTIONS

Before you sign this form please ask any questions on any aspect of this study that is unclear to you. You may take as much time as necessary to think it over.

# AUTHORISATION

I am making a decision about whether or not to participate in this study. My signature indicates that I have read and understood the information provided above, have had all my questions answered, and I have decided to participate.

The date I sign the document to enrol in this study, that is, today’s date, MUST fall between the dates indicated on the approval stamp affixed to each page. These dates indicate that this form is valid when I enrol in the study but do not reflect how long I may participate in the study. Each page of this Informed Consent Form is stamped to indicate the form’s validity as approved by the MRCZ.

_____________________________ _______________

Name of Research Participant (please print) Date

_____________________________ AM ___________

Signature of Participant PM Time

______________________ ______________________ _____

Names of MOHCW Staff witnessing/obtaining Signature of Staff Obtaining Date

Consent

**STATEMENT OF CONSENT TO BE AUDIOTAPED**

I understand that audio recordings will be taken during the SMS input session and the study interviews. *(For the statement below, please choose YES or NO by inserting your initials in the relevant box)*

- I agree to being audio recorded Yes

No

**______________________________ _________ ______ ______________**

Name of Participant (please print) Signature Date

**YOU WILL BE GIVEN A COPY OF THIS CONSENT FORM TO KEEP.**

If you have any questions concerning this study or consent form beyond those answered by the investigator, including questions about the research, your rights as a research subject or research related injuries; or if you feel that you have been treated unfairly and would like to talk to someone other than a member of the research team, please feel free to contact the Medical Research Council of Zimbabwe on telephone XXXXXXX
